# The Transition from the Fowler–Nordheim Regime to the Space-Charge-Limited Current Regime in the Case of a Pointed Nanometric Emitter †

**DOI:** 10.3390/mi17020269

**Published:** 2026-02-20

**Authors:** Dimitrios E. Karaoulanis, John P. Xanthakis

**Affiliations:** Electrical and Computer Engineering Department, National Technical University of Athens, 15700 Athens, Greece

**Keywords:** field emission, space-charge-limited currents, generalized Fowler–Nordheim method

## Abstract

We examine the transition from the Fowler–Nordheim (FN) field emission regime to the space-charge-limited (SCLC) regime in the case of a pointed nanometric emitter with radius of curvature R ≥ 5 nm, for which the traditional FN equations do not hold. To accomplish this, we use the generalized FN equation for the emission law and the “time of flight” methodology to solve the equations of motion. Taking advantage of the fact that emission from emitters with R = a few nm takes place primarily along the emitter axis, we approximate the paths as linear, provided that the anode is at a far distant position from the cathode. In the approach to the SCLC regime, the calculated currents for emitters with R = a few nm may differ by orders of magnitude, compared to the currents of planar emitters, for the a given fixed anode–cathode separation D. Differences of even greater orders of magnitude are obtained for these currents when R is fixed and D is varied. Furthermore, the variation of currents with D is heavily dependent on R. However, for emitters with R ≥ 25 nm, no appreciable differences in current are observed, compared to the results obtained using the planar theory. An explanation for the observed trends is given.

## 1. Introduction

The transition from the regime of field emission (FE) to the regime of space-charge- [1], despite the fact that analysis of both regimes dates back to the beginning of the previous century. The theory of FE was initially formulated in 1929 by Fowler and Nordheim (FN) [2], who ignored the image potential effects. SCLC was first formulated in 1911 by Child [3], who simplified the problem by assuming that the electron field is zero at the emitting surface. These simplifications in analysis in the two regimes were rectified for a planar emitting surface (a prerequisite for both initial theories) by Murphy and Good (MG) [4] for the FE regime in 1956, and by Barbour et al. [5] in 1953 for the SCLC regime, the latter also being called the Child–Langmuir (CL) regime or law.

However, modern emitters are far from planar, and they exhibit strong non-linearities in the electric field around their apex, making either the FN theory or the MG theory non-valid. The question then arises as to what extent the findings of Lau et al. [1] are affected by the different emission laws for current in the case of curved surfaces. Before we tackle this question, we note that the theories of both FE and SCLC have not remained stagnant since the 1950s. K.L. Jensen unified the thermionic and FE regimes [6,7,8], producing an equation valid in both cases, and Y.Y. Lau [9,10,11] extended the SCLC regimes to emitters with more than one dimension (1D). L. K. Ang and co-workers tackled the FE theory in the case of two-dimensional structures [12,13] while Forbes and coworkers devised new methods for comparing experiment to theory in the case of large-R emitters [14,15]. Furthermore, Kyritsakis and Xanthakis [16] generalized the FN equation for metallic emitters with curvature as low as radius R = 5 nm. We also note that Darr et al. [17] unified the transition of a planar (i.e., large R) diode to both the Mott–Gurney and CL regimes. In this paper we deal exclusively with the transition to the CL regime.

This generalized FN equation can be used to answer the above-mentioned problem regarding the FE-SCLC transition. It contains a second-order correction to the original FN linear electrostatic potential, and a spherical image potential instead of the planar one used by FN. The integral representing the resulting correction to the Gamow exponent— equal to deltaG2, due to this second-order potential term—can be exactly expressed in terms of the elliptic functions of the first and second kind, K(y) and E(y), leading to a correction to the usual FN v(y) function—denoted by ω(y)—for which an analytic expression is derived (see Equation (7) below). This exact second-order correction of the Gamow exponent deltaG2 hence leads to an algebraic expression for the current density. We note that there is also the method of Biswas and Ramachandran (BR) [18,19,20], which contains corrections up to third order in the electrostatic potential; however, a simple analytic expression for the current from these third-order corrections is not available as in the second-order corrections, and evaluation of the current requires a lot of numerical work. The corresponding deltaG3 has to be evaluated numerically. The increase in the work load due to the numerical calculations is not worth the increased accuracy in current density. The second-order corrections of the Kyritsakis and Xanthakis method already cause orders-of-magnitude differences to the FN results using a simple expression for the current and, furthermore, ccount well for the scaling properties of the field emission diodes [21]. These features are enough for the examination of the FN-SCLC transition. The BR method would give greater accuracy but at the cost of a heavier work load compared to the present.

Our chosen approach requires further justification. As the surface of the emitter starts deviating from the planar symmetry, the electron paths also bend, and the original theory of Lau et al. is no longer applicable. However, as the curvature of the emitter surface increases, the paths of the electrons converge towards the emitter axis [22]. If the anode is placed at a large distance from the cathode—large, that is, with respect to the emitter height—then the action of both the emitter nanometric curvature and the applied voltage will produce linear paths in the space along the axis of the emitter, where the majority of the current is concentrated. Similar arguments were also used in [18], in which third-order terms were calculated. Thus, the error involved in the estimation of the current can be ignored, and the 1D theory of Lau et al. can be used to study the transition.

## 2. Method

Because the method is well documented in [1], only a brief description will be given here, and the emphasis will be placed on describing how the use of a different emission law changes the equations to be solved. The basic ingredient of the time-of-flight method in [1] is the transformation of the Poisson equation from the spatial x-domain to the time t-domain by the relation dx = υdt, where υ is the velocity. The resulting equation is:

(1) $$\frac{d^{2}\upsilon }{dt^{2}}=\frac{eJ}{m\varepsilon_{0}}$$

where J is the current density and the remaining symbols have their conventional meaning. If this equation is integrated twice, it gives:

(2) $$\upsilon \left(t\right)=\frac{eJ}{2m\varepsilon_{0}}t^{2}+\frac{eE}{m}t+\upsilon_{0}$$

(3) $$x\left(t\right)=\frac{eJ}{6m\varepsilon_{0}}t^{3}+\frac{eE}{2m}t^{2}+\upsilon_{0}t\;$$

where E is the electric field at the surface (apex) of the emitter, and υ_0_ is the initial velocity. Because emission occurs by tunneling, υ_0_ = 0 is therefore a good approximation, though not exactly correct. Because E is the electric field at the emitter surface, J(E) is the current density at t = 0, as is implied by the structures of Equations (2) and (3).

If the time of flight of an electron is T, and V is the applied potential, then x(T)=D, the anode–cathode distance, and

(4) $$\upsilon \left(T\right)=v=\sqrt{\frac{2eV}{m}}$$

Upon defining the following:

$$k_{1}=\frac{eJ(E)}{6m\varepsilon_{0}},\;\;\;\;\;k_{2}=\frac{eE}{2m}.$$

Equations (2) and (3) take the following simple form:

(5) $$\upsilon =3k_{1}T^{2}+2k_{2}T$$

(6) $$D=k_{1}T^{3}+k_{2}T^{2}$$

Note that both k_1_ and k_2_ depend only on the electric field E. We can now solve for T from Equation (5) to obtain T as a function of E and υ. T = f(E,υ), provided that the emission law J(E) is known. Then, substituting in (6), we can obtain the velocity υ on arrival at the anode as a function of the electric field E. Finally, using (4), we can determine the relation between E and V.

The modifications that one obtains if the generalized FN equation J(E) for nanometric emitters is used in (3) and (4) are the subject of this paper. The emission law J(E) for nano-emitters with a radius of curvature R down to 5 nm is given by the following Equations (7) and (8), which use the spherical image interaction:

(7) $$J\left(E\right)=A(E)exp(-b\frac{W^{\frac{3}{2}}}{E}(v\left(y\right)+\frac{W}{eER}\omega \left(y\right))$$

(8) $$A\left(E\right)=aW^{-1}E^{2}{\left(t\left(y\right)+\frac{W}{eER}\psi \left(y\right)\right)}^{-2}$$

where W is the work function, a and b are the first and second FN constants, y is the usual variable of FE theory and v(y), t(y) are the usual elliptic functions of FE theory. ω(y) and ψ(y) are two new functions that give the corrections to the FN equation. These are defined as follows:

$$\omega \left(y\right)=\frac{4}{5}-\frac{{7y}^{2}}{40}-\frac{y^{2}ln(y)}{100}$$

$$\psi \left(y\right)=\frac{4}{3}-\frac{y^{2}}{500}-\frac{y^{2}ln(y)}{15}$$

A preliminary summary of the present paper was given at IVNC25 [23].

## 3. Results

We present results for three judicially chosen values of R and an anode–cathode distance D >> R. The value R = 50 nm is chosen as an upper limit for the generalized FN [12] equation because above this value no difference in current density is observed in (7) and (8), compared to the traditional MG formula. The value of R = 5 nm is the one below which (7) and (8) no longer hold. Actually, small errors already appear at R = 5 nm. The value R = 25 nm was chosen as an intermediate case between these two regimes. The approximation of straight electron paths in the case of the R = 50 nm emitter is not as good as in the case of the R = 5 nm emitter; however, we did not anticipate significant new information from the analysis of the R = 50 nm emitter because, as already stated, emitters with such low curvature follow the traditional FN law. Contrarily, in the case of the R = 5 nm emitter, for which the above approximation holds much better, significant new information will be presented.

**Figure 1:**
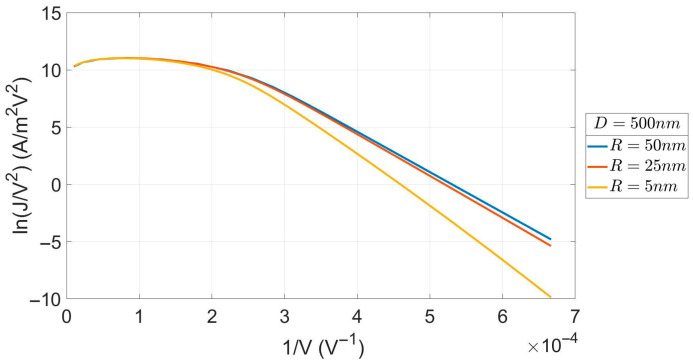
FN plots in the approach to the SCLC regime for 3 values of R.

Figure 1 gives our calculated FN plots (in the usual ln(J/V^2^) vs. 1/V form). It is observed that current density from the R = 5 nm emitter may differ by orders of magnitude from the MG results. However, this difference is annihilated as the emitter enters the SCLC regime (the far right dotted part of the diagram). On the contrary, no appreciable difference from the MG results is shown in the case of the R = 25 nm emitter. Overall, the form of the plot is retained irrespective of the curvature of the emitter, but the approach to the SCLC regime gets slower as the emitter becomes sharper. We also note a small decline in the SCLC current density at very high voltages. This has also been seen in the calculations of Forbes [24]. However, it is not seen experimentally. Forbes [24] has argued that the experimental fields are not strong enough to portray this feature.

**Figure 2:**
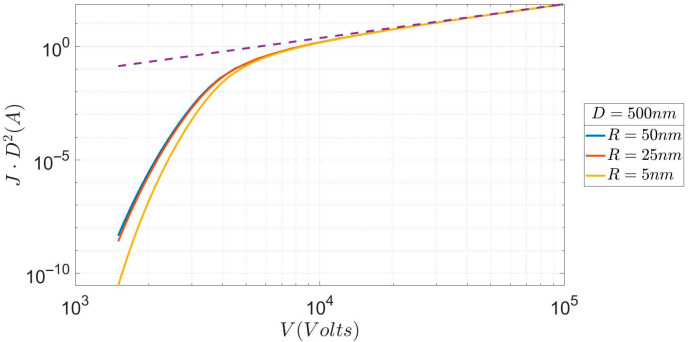
Current (normalized to D^2^) vs. voltage for 3 values of R over an extended voltage, so as to show the convergence with the Child-Langmuir SCLC regime (dotted line).

A more informative picture of the transition is given in Figure 2, where the *x*-axis, the voltage, is more extended than in Figure 1 and the asymptotic convergence to the SCLC V^3/2^ law can be clearly seen (dotted line). The characteristics hold for the same three values of R and D as in Figure 1. The comments made to Figure 1 are also applicable here. Note that although the differences in current density seem small to the eye, because the *y*-axis spans ten orders of magnitude, they are substantial in the approach to the SCLC regime. Furthermore, compared to the corresponding figure of Lau et al. [1], the approach to the SCLC regime is much smoother, i.e., the characteristics are not so “vertical” in the approach to the SCLC regime. This difference is due to both the omission of image effects in [1] and the small curvature of the emitter studied here. Next, we examine the effects of different values of D, for which large variations in the current density are observed.

**Figure 3:**
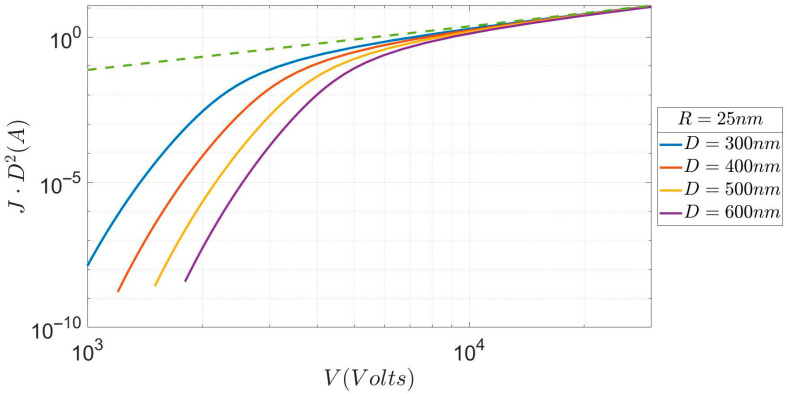
Current (normalized to D^2^) vs. voltage for an emitter with R = 25 nm for several anode–cathode distances D. The dotted line represents the Child-Langmuir law of the SCLC regime.

**Figure 4:**
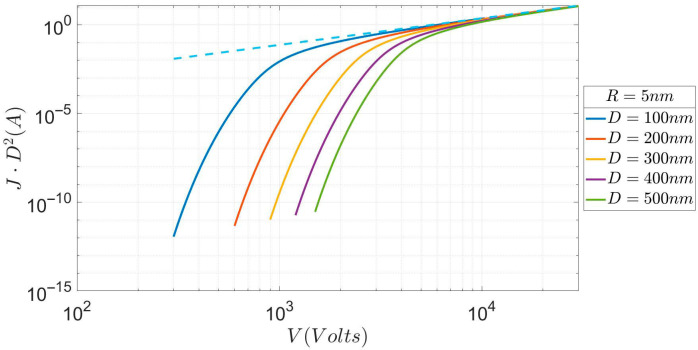
Current (as in Figure 3) vs. voltage for an emitter of R = 5 nm and several values of D.

Figure 3 gives the variation in the I-V characteristics of an R = 25 nm emitter for various values of D (much greater than R). It can be seen that the difference in current density between D = 300 nm and D = 600 nm can be between three and five orders of magnitude, depending on the value of the applied voltage V. The information contained in Figure 3 is not new, in the sense that qualitatively similar characteristics were obtained by Lau et al. [1] for a planar diode. Our new and original contribution to the subject will appear when we compare Figure 3 with the following Figure 4, which contains the I-V characteristics of an R=5nm emitter for various values of D.

Again, as in Figure 3, the difference in current density between, say, the D = 200 nm characteristic and the D = 500 nm one, may be four to six (or more) orders of magnitude, depending on the value of the voltage V. However, the most noticeable difference from Figure 3 is the steep increase in current density here. The following Table 1 shows this. The cause of this increased steepness can be easily understood. We remind the reader that the R = 50 nm characteristic in Figure 2 (which is almost the same as for a planar diode) is smoother (slower increase in current) than the corresponding planar obtained by Lau et al. [1]. The cause of this difference is attributed to the use of the triangular barrier in [1], i.e., to a higher barrier than the image rounded barrier here. The same mechanism applies (as an explanation) to the difference observed between Figure 3 and Figure 4. As R decreases, the second-order correction to the electrostatic potential becomes more important because it varies as 1/R. However, this term has the opposite sign compared to the linear term, resulting in a slower decrease in the tunneling potential with distance. This effectively extends the barrier length, resulting in a lower transmission coefficient because the latter is a path integral in real space along the tunneling region (see [16] for more details). Hence, the cause of this difference is the increase in the barrier strength as R is decreased (because the path integral representing it is taken along a wider range).

## 4. Conclusions

In this work, we examine the transition from the FN regime to the SCLC regime in the case of a pointed nanometric emitter described by a radius of curvature R. When R = a few nm, a difference in current of three-to-five orders of magnitude is obtained in the approach to the SCLC regime, compared to the results obtained with the Murphy–Good (or FN) theory, when the anode–cathode separation D is fixed and R is varied. When R is fixed and D is varied, differences in current of even greater orders of magnitude are obtained. The observed trends are explained in terms of the corresponding changes in the tunneling potentials.

## Figures and Tables

**Table 1:** Differences in normalized current for emitter with various radii of apex at 2000 V.

	R (nm)	D (nm)	J·D^2^ at 2000 V (A)
Figure 3	25	300	~5 × 10^−3^
Figure 3	25	400	~1 × 10^−4^
Figure 3	25	500	~7 × 10^−5^
Figure 4	5	300	~1 × 10^−3^
Figure 4	5	400	~8 × 10^−4^
Figure 4	5	500	~1 × 10^−7^

## Data Availability

All data required for this work are available in this paper.
